# Intrarenal renin–angiotensin system activation and macrophage infiltrations in pediatric chronic glomerulonephritis

**DOI:** 10.1007/s00467-023-06026-5

**Published:** 2023-05-25

**Authors:** Tomoki Hattori, Keisuke Fujioka, Takashi Nagai, Shuji Kondo, Shoji Kagami, Masahiro Hirayama, Maki Urushihara

**Affiliations:** 1 Department of Pediatrics, Tokushima University Graduate School of Biomedical Sciences, Kuramoto-cho 3-18-15, Tokushima, Tokushima, 770-8503 Japan; 2 Department of Pediatrics, Mie University Graduate School of Medicine, Mie, Japan

**Keywords:** Renin–angiotensin system, Angiotensinogen, Pediatric chronic glomerulonephritis, CD68, Monocyte chemoattractant protein-1

## Abstract

**Background:**

The current study tested the hypothesis that urinary angiotensinogen (UAGT) and urinary monocyte chemoattractant protein-1 (UMCP-1) levels provide a specific index of intrarenal renin–angiotensin system (RAS) status and the degree of infiltration of macrophages associated with RAS blockade and immunosuppressant treatment in pediatric patients with chronic glomerulonephritis.

**Methods:**

We measured baseline UAGT and UMCP-1 levels to examine the correlation between glomerular injury in 48 pediatric chronic glomerulonephritis patients before treatment. Furthermore, we performed immunohistochemical analysis of angiotensinogen (AGT) and CD68 in 27 pediatric chronic glomerulonephritis patients treated with RAS blockades and immunosuppressants for 2 years. Finally, we examined the effects of angiotensin II (Ang II) on monocyte chemoattractant protein-1 (MCP-1) expression in cultured human mesangial cells (MCs).

**Results:**

Baseline UAGT and UMCP-1 levels positively correlated with urinary protein levels, scores for mesangial hypercellularity, rate of crescentic formation, and expression levels of AGT and CD68 in renal tissues (*p* < 0.05). UAGT and UMCP-1 levels were significantly decreased after RAS blockade and immunosuppressant treatment (*p* < 0.01), which was accompanied by AGT and CD68 (*p* < 0.01), as well as the magnitude of glomerular injury. Cultured human MCs showed increased MCP-1 messenger ribonucleic acid and protein levels after Ang II treatment (*p* < 0.01).

**Conclusions:**

The data indicates that UAGT and UMCP-1 are useful biomarkers of the degree of glomerular injury during RAS blockade and immunosuppressant treatment in pediatric patients with chronic glomerulonephritis.

## Introduction

Activation of the renin–angiotensin system (RAS) plays a pivotal role in arterial pressure and sodium homeostasis [1]. In particular, local renal RAS activation has been reported to be deeply involved in the progression of kidney injury in patients with chronic glomerulonephritis [2]. By evaluating the level of angiotensinogen (AGT) expression in renal glomeruli and measuring urinary AGT (UAGT), it is possible to confirm the degree of activation of RAS in the renal region and estimate the disease state of chronic glomerulonephritis [3]. In our previous reports, we confirmed that AGT expression was enhanced in renal endothelial and mesangial cells (MCs) in pediatric immunoglobulin A nephropathy (IgAN) [4]. Furthermore, we showed that UAGT is an index of intrarenal RAS activation in children with IgAN [5].

Activation and infiltration of monocytes/macrophages into kidney tissues and fibrosis are involved in the progression of kidney injury along with RAS activation [6, 7]. Recently, the degree of macrophage infiltration into the renal region has been evaluated by CD68 expression, a surface marker of macrophages, in renal glomeruli and by measuring the urinary concentration of monocyte chemoattractant protein-1 (MCP-1), which is a monocyte chemoattractant [8–11]. In addition, it was suggested that RAS activation induces CD68-positive macrophages and promotes crescent formation in a rat model of crescentic glomerulonephritis [12]. However, few reports have examined RAS activation and macrophage induction in pediatric patients with chronic glomerulonephritis. In the present study, we investigated the relationship between RAS activation and macrophages in childhood chronic glomerulonephritis.

## Materials and methods

### Participants and study protocol

The study protocol was approved by the Institutional Review Board of the University of Tokushima Graduate School (1085–3). We recruited 48 pediatric patients who were diagnosed with chronic glomerulonephritis by clinical course and kidney biopsy at Tokushima University Hospital from January 1, 2014, to October 1, 2022. We enrolled pediatric patients with IgAN and Henoch–Schönlein purpura nephritis as participants with major chronic glomerulonephritis. All participants signed a consent form. When a participant was not capable of providing assent based on age, a simple oral explanation of the study was offered, and a written parental permission was obtained. At the time of study enrollment, all patients were asked to undergo repeat kidney biopsies for two years following immunosuppressive therapy (prednisolone, cyclophosphamide, or mizoribine) and RAS blockade (candesartan). A total of 27 patients underwent repeat kidney biopsy 2 years after treatment. We referred to the second biopsy period as post-treatment, and at that time all 27 patients had negative proteinuria. Additionally, all could discontinue prednisolone and cyclophosphamide before the second biopsy but continued RAS blockade and mizoribine. Clinical parameters, including sex, age, height, body weight, and blood pressure, laboratory parameters, including serum concentrations of sodium, potassium, and creatinine, and urinary concentrations of protein and creatinine, were determined at the time of biopsy. The estimated glomerular filtration rate (eGFR) was calculated using the Modification of Diet in Renal Disease formula (eGFR = 175 × standardized serum creatinine ^–1.154^ × age ^–0.203^ × 0.741 [if Asian] × 0.742 [if female]) [13], which was found to correlate well with the glomerular filtration rate corrected for body surface area in adults [14]. Urinary concentrations of AGT were measured using a human Total AGT Assay kit (IBL, Fujioka, Japan) as previously described [15]. Additionally, we performed sandwich enzyme-linked immunosorbent assays (ELISA) for MCP-1 in urine in accordance with the manufacturer’s specifications (Boster Bio, Pleasanton, CA, USA).

### Histological study

For light microscopic examination, biopsied tissues were fixed in 10% buffered formalin and embedded in paraffin. Paraffin sections (3 µm) were stained with periodic acid-Schiff reagent. All glomeruli in each section (usually 10–37) were coded and read by two independent observers who were blinded to the clinical data. Consistent with previous studies [16], the rate of mesangial hypercellularity was evaluated by the proportion of glomeruli with mesangial cell proliferation among the observed glomeruli, with over four mesangial cells per peripheral lobule. The number of crescentic glomeruli was calculated as the percentage of glomeruli with crescents (including cellular or fibrocellular) among the observed glomeruli. To evaluate the level of tubulointerstitial change, three to five fields of the cortical interstitium in each section were examined. In each field, tubulointerstitial changes, such as interstitial fibrosis and tubular atrophy, were evaluated as percentages of tissue. Furthermore, we graded each according to pathological classification, Oxford classification [17], and the International Study of Kidney Disease in Children (ISKDC) grading [18].

### Immunohistochemistry

Immunohistochemistry for AGT or CD68 was performed using formalin-fixed paraffin-embedded kidney sections. Primary antibody against human AGT (11,992–1-Ap) was purchased from Proteintech (1:300; Rosemont, IL, USA). Anti-CD68 (ab955) antibody for macrophage surface markers was obtained from Abcam (1:50; Cambridge, MA, USA). Sections (3 µm) were incubated with primary antibodies overnight at 4 °C. After rinsing, the sections were incubated with biotinylated anti-rabbit IgG (Vector Labs, Burlingame, CA, USA) using anti-AGT antibody. The other sections that used anti-CD68 antibody were incubated with biotinylated anti-mouse IgG (Vector Labs). After rinsing, the sections were incubated with an avidin–biotin-peroxidase complex (ABC Elite; Vector Labs), followed by 3,3′-diaminobenzidine (Dojindo, Kumamoto, Japan). Each section was counterstained with Mayer’s hematoxylin (Wako, Tokyo, Japan), dehydrated, and coverslipped. The fraction of immunoreactive area (brown) was measured using EIS-Elements software (Nikon Corporation, Tokyo, Japan). For each glomerulus, the immunoreactive area (brown) was automatically calculated by the software (EIS-Elements, Nikon Corporation, Tokyo, Japan), and this affected area was, in turn, divided by the total area of the glomerulus. At least three equatorially sectioned glomeruli were examined on each slide.

For double-staining experiments, frozen sections (3 µm) were fixed in acetone and incubated with rabbit anti-AGT antibody (11,992–1-Ap; Proteintech) and mouse anti-CD68 antibody (ab955; Abcam) overnight at 4 °C, followed by fluorescein-isothiocyanate-labeled anti-rabbit IgG and rhodamine-labeled anti-mouse IgG (Jackson ImmunoResearch Laboratories, West Grove, PA, USA).

### Cell culture

Cultured human MCs derived from normal human kidneys were purchased from ScienCell Research Laboratories (Carlsbad, CA, USA). Human MCs were maintained in commercially recommended growth medium (Sigma-Aldrich, St. Louis, MO, USA), and were used at the seventh to tenth passages in our experiments. At approximately 70–80% confluence, human MCs were arrested by incubation in serum-free medium for 48 h and then stimulated with Ang II (Wako) at increasing concentrations (0, 10^–11^, 10^–9^, 10^–7^ M) for 2–24 h, to detect MCP-1 messenger ribonucleic acid (mRNA) or protein, respectively. In addition, human MCs were treated with Ang II type 1 receptor blocker (ARB) (Sigma-Aldrich) at 10^–4^ M concentration for 1 h followed by stimulation with Ang II at 10^–7^ M concentration for 2–24 h to detect MCP-1 mRNA or protein. We used real-time polymerase chain reaction (RT-PCR) to examine MCP-1 mRNA, and sandwich ELISA for the MCP-1 protein assay. Cells were harvested for RNA extraction and total cell extract preparation.

### Quantitative RT-PCR

Total RNA was extracted using a commercially available kit (Qiagen, Hilden, Germany). A total of 1 μg of RNA was reverse transcribed using the High-Capacity cDNA Reverse Transcription Kit (Applied Biosystems, Foster City, CA, USA) according to the manufacturer’s instructions. The TaqMan Gene Expression Master Mix and TaqMan Gene Expression Assays (Applied Biosystems) were used for PCR. Amplification and detection were performed using a StepOnePlus Real-Time PCR System (Applied Biosystems). TaqMan Gene Expression Assays for each gene were purchased from Applied Biosystems for the following human genes: MCP-1 (Hs00234140_m1) and glyceraldehyde-3-phosphate dehydrogenase (Hs02786624_g1).

### Statistical analysis

Pearson and Spearman correlation coefficients were used for parametric and nonparametric data, respectively. The Wilcoxon signed-rank test was used to perform paired comparisons before and after the treatment. The mRNA expression or protein synthesis of MCP-1 in cultured cells was analyzed using a t-test. All data are presented as mean ± standard error of the mean. Statistical significance was set at *P* < 0.05. All computations, including data management and statistical analysis, were performed using the JMP software (SAS Institute, Cary, NC, USA).

## Results

### Demographics, laboratory data and histological findings

**Fig. 1:**
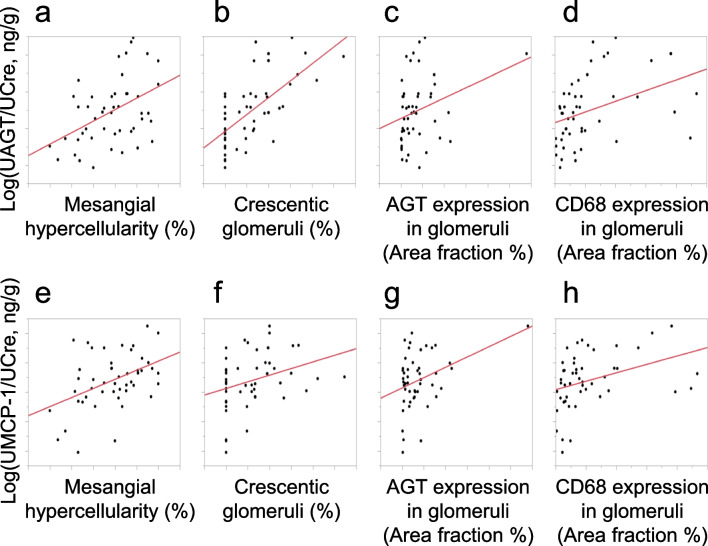
Plots of correlations between (**a**) log (UAGT/UCre) and mesangial hypercellularity, (**b**) log (UAGT/UCre) and crescentic glomeruli, (**c**) log (UAGT/UCre) and AGT expression in glomeruli, (**d**) log (UAGT/UCre) and CD68 expression in glomeruli, (**e**) log (UMCP-1/UCre) and mesangial hypercellularity, (**f**) log (UMCP-1/UCre) and crescentic glomeruli, (**g**) log (UMCP-1/UCre) and AGT expression in glomeruli, (**h**) log (UMCP-1/UCre) and CD68 expression in glomeruli, in pediatric patients with CGN. log (UAGT/UCre), logarithmically transformed urinary angiotensinogen/urinary creatinine ratio; log (UMCP-1/UCre), logarithmically transformed urinary monocyte chemoattractant protein-1/urinary creatinine ratio

The demographic and baseline laboratory data of the included subjects are summarized in Table 1 whereas pathological classifications are shown in Table 2. Table 3 shows the single-regression analyses for logarithmically transformed UAGT/creatinine ratio (log [UAGT/UCre]) with clinical parameters. Log (UAGT/UCre) levels were significantly positively correlated with urinary protein–creatinine ratio (UPro/UCre), rate of mesangial hypercellularity, rate of crescentic glomeruli, and glomerular expression levels of AGT and CD68 in kidney tissue. However, log (UAGT/UCre) levels did not correlate with the eGFR, interstitial fibrosis, and tubular atrophy. Table 3 also shows the single-regression analyses for logarithmically transformed urinary MCP-1/creatinine ratio (log [UMCP-1/UCre]) with clinical parameters. Log (UMCP-1/UCre) levels were significantly positively correlated with UPro/UCre, rate of mesangial hypercellularity, rate of crescentic glomeruli, and glomerular expression levels of AGT and CD68 in kidney tissue. However, log (UMCP1/UCre) levels did not correlate with the eGFR, interstitial fibrosis, and tubular atrophy. Additionally, Fig. 1 shows plots of positive correlations of these data. Table 4 presents the type of immunosuppressants (prednisolone, cyclophosphamide, or mizoribine) and RAS blockade (candesartan) used by the 27 patients who underwent repeat kidney biopsy 2 years after treatment. Baseline and post-treatment indices of blood pressure and laboratory data for the 27 patients who underwent repeat kidney biopsies are shown in Table 5. The UPro/UCre was significantly lower post-treatment. There were no significant changes in systolic or diastolic blood pressure or serum concentrations of sodium, potassium or eGFR.

**Table 1:** Baseline characteristics of 48 patients with chronic glomerulonephritis

Parameters	Data (mean ± standard deviation)
Sex, male/female	31/17
Age, year, median (range)	10.8 (2.8–17.4)
Diagnosis of CGN	IgAN: HSPN = 33: 15
Height, cm	135.88 ± 22.94
Body weight, kg	34.26 ± 15.02
BMI	17.54 ± 3.04
Systolic blood pressure, mmHg	105.0 ± 10.7
Diastolic blood pressure, mmHg	63.1 ± 8.2
Serum Cre, mg/dL	0.43 ± 0.15
UPro/UCre, g/g, median [IQR]	0.52 [0.14–1.66]

CGN; chronic glomerulonephritis, IgAN; immunoglobulin A nephropathy, HSPN; Henoch-Schönlein purpura nephritis, BMI; body mass index, Cre; creatinine, UPro/UCre; urinary protein-creatinine ratio, IQR; interquartile range

**Table 2:** Baseline histological classification of 48 pediatric patients

Oxford Classification	M0 / M1	E0 / E1	S0 / S1	T0 / T1 / T2	C0 / C1 / C2
N (= 33)	12 / 21	20 / 13	15 / 18	33 / 0 / 0	15 / 18 / 0
ISKDC grade	II	IIIa	IIIb	IV	V
N (= 15)	3	4	8	0	0

M; mesangial hypercellularity, E; endocapillary hypercellularity, S; segmental glomerulosclerosis, T; tubular atrophy/interstitial fibrosis, C; crescents, ISKDC; the international study of kidney disease in children

**Table 3:** Urinary angiotensinogen and urinary monocyte chemoattractant protein-1 correlation between laboratory data and histological findings in patients with chronic glomerulonephritis

	Urinary AGT		Urinary MCP-1	
Parameters	*R* values	*P* values	*R* values	*P* values
e-GFR	0.1348	0.3610	0.0682	0.6452
UPro/UCre	0.5428	< 0.0001*	0.4644	0.0009*
Mesangial hypercellularity	0.4554	0.0011*	0.4353	0.0020*
Crescentic glomeruli	0.6635	< 0.0001*	0.3158	0.0288*
Interstitial fibrosis	0.1877	0.2014	0.1122	0.4477
Tubular atrophy	0.1424	0.3344	0.0173	0.9072
AGT expression in glomeruli CD68 expression in glomeruli	0.2944	0.0422*	0.3593	0.0121*
	0.3816	0.0074*	0.3620	0.0115*

angiotensinogen; AGT, monocyte chemoattractant protein-1; MCP-1, eGFR; estimated glomerular filtration rate, UPro/UCre; urinary protein-creatinine ratio, *; *P* < 0.05

**Table 4:** Immunosuppressive treatment for CGN

Treatments	No. *N* = 27
CS + MPT + POCY + ARB	2
CS + POCY + MZR + ARB	3
CS + POCY + ARB	11
CS + MZR + ARB	11

CGN; chronic glomerulonephritis, CS; corticosteroid, MPT; methylprednisolone pulse therapy, POCY; peroral cyclophosphamide, ARB; angiotensin II type 1 receptor blocker, MZR: mizoribine

**Table 5:** Changes in blood pressure, laboratory data and histological findings

Parameters	*Baseline N* = *27*	*Posttreatment N* = *27*	Change	*P* values
sBP, mmHg	103.7 ± 10.8	104.0 ± 8.06	0.33 ± 2.13	0.8770
dBP, mmHg	61.4 ± 8.65	58.5 ± 6.75	–2.93 ± 2.37	0.2281
Serum Na, mEq/L	140.8 ± 2.04	140.6 ± 1.83	–0.22 ± 0.52	0.6733
Serum K, mEq/L	4.16 ± 0.33	4.02 ± 0.36	–0.13 ± 0.09	0.1433
eGFR, ml/min	123.6 ± 23.7	122.4 ± 24.4	–1.24 ± 5.74	0.8307
UPro/UCre, g/g	2.49 ± 4.03	0.13 ± 0.16	–2.36 ± 0.77	0.0050*
Mesangial hypercellularity, %	69.1 ± 22.5	36.0 ± 23.3	–33.1 ± 4.92	< 0.0001*
Crescentic glomeruli, %	7.78 ± 6.96	1.10 ± 3.44	–6.68 ± 1.44	< 0.0001*
Interstitial fibrosis, %	2.60 ± 3.21	2.60 ± 5.94	–0.00 ± 1.25	0.5000
Tubular atrophy, %	2.04 ± 3.74	2.59 ± 3.5	0.55 ± 0.90	0.5417
AGT expression in glomeruli, area fraction %	4.82 ± 6.00	2.28 ± 3.21	–2.54 ± 0.67	0.0008*
CD68 expression in glomeruli, area fraction %	2.24 ± 3.34	0.24 ± 0.34	–2.00 ± 0.64	0.0043*

SBP; systolic blood pressure, DBP; diastolic blood pressure, eGFR; estimated glomerular filtration rate, UPro/UCre; urinary protein-creatinine ratio, AGT; angiotensinogen, *; *P* < 0.05

### UAGT and UMCP-1 levels and histological findings

**Fig. 2:**
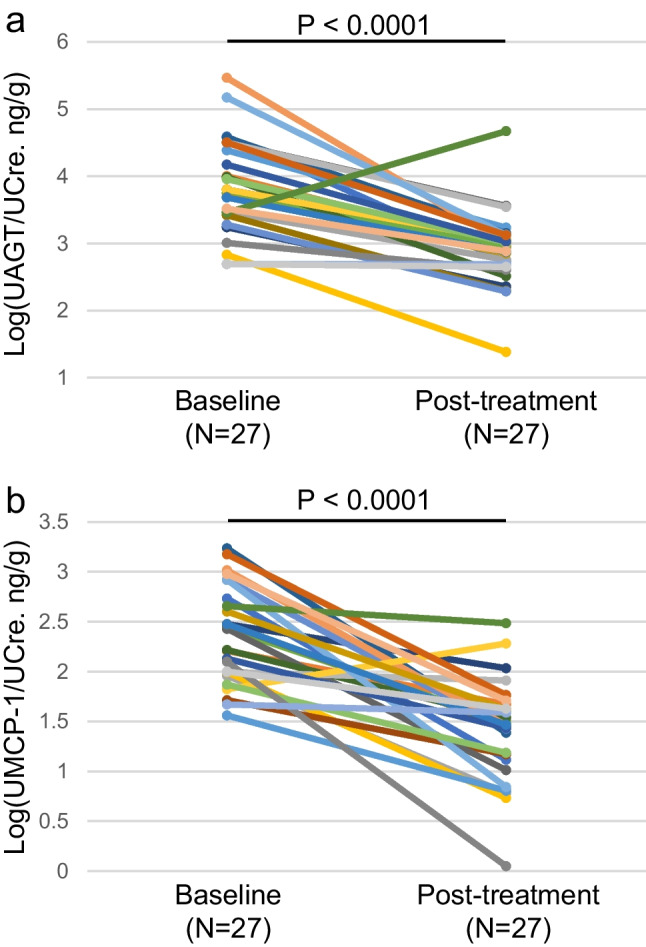
(**a**) Changes in logarithmically transformed UAGT/UCre ratio (log [UAGT/UCre]) after treatment in pediatric patients with CGN. (**b**) Changes in the logarithmically transformed UMCP-1/UCre ratio (log [UMCP-1/UCre]) in pediatric patients with CGN after treatment. Each marker and line represents an individual participant in the study. UAGT, urinary angiotensinogen; UCre, urinary creatinine; CGN, chronic glomerulonephritis; UMCP, urinary monocyte chemoattractant protein-1

**Fig. 3:**
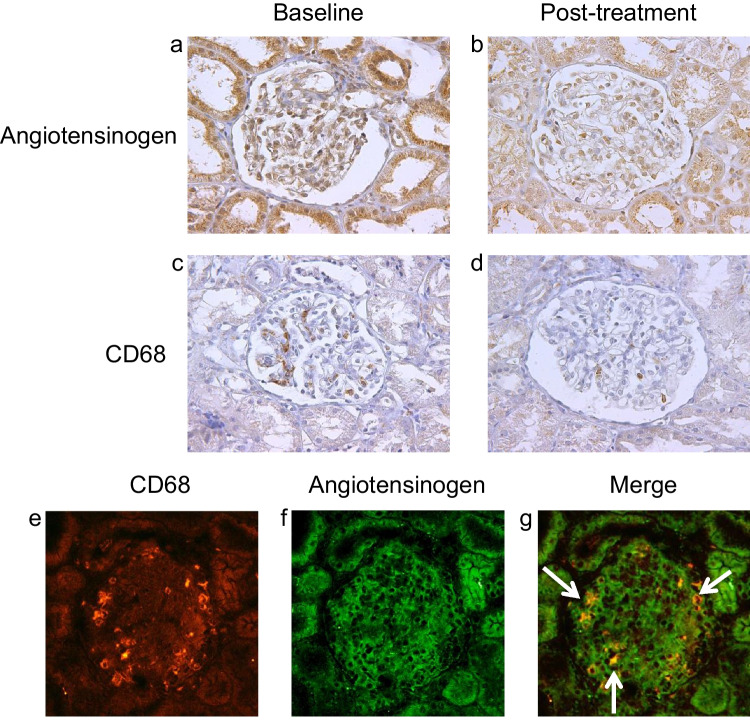
(**a**, **b**) Representative images of AGT, and (**c**, **d**) CD68 in pediatric patients with IgAN at (**a**, **c**) baseline vs. (**b**, **d**) posttreatment. Immunosuppressive therapy decreased the immunoreactivity of AGT and CD68 in renal tissues from pediatric patients with IgAN. Immunofluorescence staining of the glomeruli in pediatric patients with IgAN at pretreatment. (**e**) Double staining for CD68 (red) and (**f**) angiotensinogen (green). Staining for angiotensinogen was observed on (**g**) CD68 positive macrophage (yellow, white arrow). AGT, angiotensinogen; IgAN, immunoglobulin A nephropathy

Figure 2 illustrates the change in log (UAGT/UCre) (Fig. 2a) and log (UMCP-1/UCre) (Fig. 2b). The levels of log (UAGT/UCre) were significantly decreased at the end of the study (3.86 ± 0.71 ng/g vs. 2.90 ± 0.56 ng/g). There were also significant decreases in log (UMCP-1/UCre) levels in patients treated with immunosuppressant and RAS blockade (2.38 ± 0.47 ng/g vs. 1.41 ± 0.51 ng/g). The rate of mesangial hypercellularity, crescentic glomeruli, and glomerular expression levels of AGT and CD68 in glomeruli were lower at the end of the study than at baseline (Table 5, Fig. 3a-c).

To investigate the localization of AGT and CD68 in the kidney tissue of patients with IgAN, double immunofluorescence staining was performed. Double immunostaining showed that CD68 was positive in the macrophages of the glomeruli (Fig. 3e). AGT was primarily detected in glomerular endothelial cells, to a lesser extent in MCs, and only faintly in podocytes (Fig. 3f), which is consistent with a recent report [4]. AGT was localized not only in glomerular cells but also in macrophages infiltrating the glomeruli (Fig. 3g).

### Effect of Ang II on MCP-1 mRNA and protein levels in cultured human MCs

**Fig. 4:**
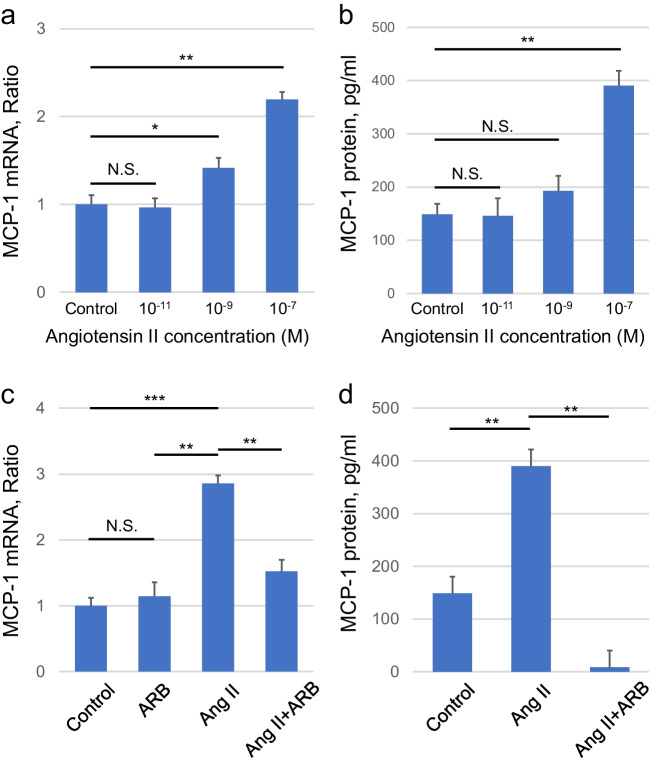
Effect of Ang II on cultured human MCs. (**a**) Human MCs were incubated for 2 h with vehicle control or indicated dose of Ang II, extracted, and analyzed for MCP-1 mRNA levels by RT-PCR. (**b**) Human MCs were incubated for 24 h with vehicle control or indicated dose of Ang II, extracted, and analyzed for MCP-1 protein levels by sandwich ELISA. Human MCs were pretreated with ARB and subsequently stimulated with Ang II. MCP-1 expression was measured by (**c**) quantitative RT-PCR and (**d**) sandwich ELISA. Data are mean ± SEM. * *P* < 0.05, ** *P* < 0.01, and *** *P* < 0.001 between groups as indicated. Ang II, angiotensin II; MCs, mesangial cells; ARB, Ang II type 1 receptor blocker; MCP-1, monocyte chemoattractant protein-1; RT-PCR, real-time polymerase chain reaction; ELISA, enzyme-linked immunosorbent assays; SEM, standard error of the mean; N.S., not significant

The regulation of MCP-1 expression by human MCs and the possible role of RAS-MCs-MCP-1 in the disease progression of chronic glomerulonephritis were examined using cultured human MCs. When the response of MCP-1 transcript to Ang II dose was investigated after incubating for 2 h, a dose-dependent increase between 10^–11^ and 10^–7^ M was observed (Fig. 4a). Consistent with the RT-PCR analysis results, a dose-dependent increase in human MC-MCP-1 protein to Ang II stimulation was observed between 10^–11^ and 10^–7^ M by ELISA (Fig. 4b). Following the incubation of quiescent MCs with 10^–7^ M Ang II, quantitative PCR revealed an increase in MCP-1 mRNA levels. ARB treatment reduced the elevated MCP-1 expression (Fig. 4c), which were confirmed by ELISA (Fig. 4d).

## Discussion

Recent human studies have demonstrated the involvement of UAGT levels in RAS activation and the development of cardiovascular disease, hypertension, and kidney disease [1, 3]. In contrast, macrophage infiltration in progressive glomerulonephritis was first reported by Atkins et al. in 1976 [6]. Since then it has been found that infiltration and accumulation of macrophages into the glomeruli and interstitium are observed in advanced chronic kidney disease with various pathological conditions [19]. Many signaling pathways and hormonal systems have been implicated in macrophage activation and disease progression in all organs of the body. Among the various signaling pathways that induce macrophages, RAS is considered an important factor [20]. However, few reports have investigated the interaction between RAS activation and macrophage infiltration in kidney diseases. In a previous report, we reported that administration of an Ang II receptor blocker to rats, a model of crescent-forming glomerulonephritis inducing CD68-positive macrophages and crescent formation following MCP-1 production, attenuated the progression of crescentic glomerulonephritis [12]. Here, we investigated the relationship between RAS activation and macrophages in 48 children with chronic glomerulonephritis.

We demonstrated that UAGT levels were positively correlated with urinary protein levels, mesangial hypercellularity, crescentic glomeruli ratio, and the expression levels of AGT and CD68 in the glomeruli. Moreover, UMCP-1 levels were positively correlated with urinary protein levels, mesangial hypercellularity, crescentic glomeruli ratio, and the expression levels of AGT and CD68 in the glomeruli. We also demonstrated that UAGT and UMCP-1 levels and renal AGT and CD68 expression were decreased in pediatric patients with chronic glomerulonephritis treated with immunosuppressive therapy.

We previously tested the hypothesis that UAGT levels reflect the intrarenal RAS status in chronic glomerulonephritis during childhood. We found that UAGT levels were significantly increased in patients with chronic glomerulonephritis who were not treated with immunosuppressants compared to control subjects. Notably, patients with chronic glomerulonephritis during childhood treated with immunosuppressive therapy showed a marked attenuation of this augmentation. Thus, UAGT excretion and renal AGT expression are associated with the pathophysiology of kidney injury in human subjects, especially in pediatric patients with chronic glomerulonephritis. In the present study, the correlation analysis provided some important findings. UAGT levels were positively correlated with the glomerular expression levels of AGT in kidney tissue, mesangial hypercellularity, and crescentic glomeruli. In addition, UAGT and glomerular AGT expression levels decreased after immunosuppressive treatment. These data are compatible with previous reports and indicate that the intrarenal RAS is activated through local augmentation of AGT production in IgAN patients [4, 5]. These findings, along with our present findings, indicate that UAGT is a novel biomarker of intrarenal RAS activation in pediatric patients.

We further demonstrated that the UMCP-1 levels positively correlated with the glomerular expression levels of CD68 in kidney tissue, mesangial hypercellularity scores, and crescentic glomeruli. UMCP-1 and glomerular CD68 expression decreased after immunosuppressive treatment.

It is well known that CD68-positive macrophage infiltration in glomeruli is a major mediator in the pathogenesis of progressive glomerular injury for crescentic glomerulonephritis patients [10]. MCP-1 induces macrophage invasion, and other studies have shown that the degree of active renal lesions is associated with high sensitivity to the beneficial effects of immunosuppressive therapy on glomerular structure and proteinuria in vasculitis-associated nephritis [9]. Therefore, we believe that UMCP-1 is a useful marker for monitoring changes in intraglomerular macrophage infiltrate levels associated with glomerular injury in pediatric patients with chronic glomerulonephritis.

We also examined the correlation between RAS activation and macrophage infiltration in pediatric patients with chronic glomerulonephritis. In the present study, treatment with immunosuppressants reduced the urinary and glomerular expression levels of MCP-1 associated with reduced glomerular AGT and UAGT levels, which suggests mitigation of intrarenal RAS activation. UAGT levels positively correlated with the expression levels of CD68-positive macrophages in glomeruli, and UMCP-1 levels positively correlated with AGT expression levels in glomeruli. These findings suggest that the overexpression of MCP-1 induced by intrarenal RAS activation contributes to the development of glomerular injury in pediatric patients with chronic glomerulonephritis. In order to speculate how infiltrating macrophages in glomeruli act on the development of glomerular injury, we performed double staining of glomerular AGT and CD68 in patients with IgAN. CD68-positive cells were also stained with AGT, suggesting that CD68-positive macrophages produce AGT, which may promote intrarenal RAS activity. We have previously disclosed the mechanism and role of the relationship between RAS activation and MCP-1 production in rat MCs. Ang II stimulates MCP-1 synthesis in cultured rat MCs. In rat kidneys, MCP-1 induces macrophage infiltration into glomeruli, resulting in the development of glomerular crescent formation.

This suggests that activated or injured MCs synthesize AGT, triggering a cascade of Ang II and MCP-1 gene expression [12]. We demonstrated similar results in human MCs, and RAS blockade with ARB, which reduced the production of MCP-1 mRNA and protein.

The relatively small sample size is a potential limitation of the present study. For instance, the present study did not enroll patients with kidney dysfunction because all participants underwent biopsy no longer than one year after the onset of abnormal urinalysis, and they were picked up at an early stage of the disease. Therefore, it is difficult to further classify patients according to their urinary protein levels or pathology patterns. However, this study demonstrated that UAGT and UMCP-1 levels were significantly decreased after immunosuppressive therapy, accompanied by mesangial hypercellularity score, ratio of crescentic glomeruli, renal AGT, and CD68 expression. Furthermore, UAGT and UMCP-1 levels correlated with these parameters. These data strongly support the hypothesis that UAGT and UMCP-1 are powerful tools for determining intrarenal RAS status and associated glomerular injury, including mesangial hypercellularity and crescentic glomeruli in pediatric chronic glomerulonephritis. However, future evaluation of UAGT and UMCP-1 in prospective studies with a larger number of patients and an extended observation period is needed.

In conclusion, these data support the hypotheses that glomerular AGT expression and UAGT levels are useful for evaluating intrarenal RAS activation, and if the activation is strong, treatment with RAS inhibitors is effective. On the other hand, glomerular CD68 expression and UMCP-1 levels are used to evaluate the level of macrophage infiltration into the glomeruli, and if the infiltration and accumulation are strong, we presumed that immunosuppressive therapy is effective.

## Data Availability

Primary data is available upon request from the corresponding author.
